# TTK is a potential therapeutic target for cisplatin-resistant ovarian cancer

**DOI:** 10.1186/s13048-021-00884-z

**Published:** 2021-10-02

**Authors:** Yixuan Liu, Keyu Zhu, Xiaolin Guan, Suhong Xie, Yanchun Wang, Ying Tong, Lin Guo, Hui Zheng, Renquan Lu

**Affiliations:** 1 Department of Clinical Laboratory, Fudan University Shanghai Cancer Center, No.270, Dong’An Road, Xuhui District, Shanghai, 200032 China; 2 Department of Oncology, Shanghai Medical College, Fudan University, Shanghai, China; 3 Shanghai Ninth People’s Hospital, Shanghai JiaoTong University School of Medicine, Shanghai, China

**Keywords:** Threonine and tyrosine kinase (TTK), Cisplatin resistance, Ovarian cancer, Signaling pathway

## Abstract

**Background:**

Drug resistance and recurrence are main contributors to the poor prognosis of ovarian cancer. Cisplatin is a platinum compound which is widely used in the treatment of various solid tumors including ovarian cancer. Up to now, the mechanism of cisplatin resistance in ovarian cancer is unclear. Threonine and tyrosine kinase (TTK), an integral part of the spindle assembly checkpoint, may be a potential new target associated with chemotherapy sensitivity.

**Results:**

TTK was up-regulated in the cisplatin-resistant ovarian cancer cell line. Down-regulation of TTK could recover the sensitivity of cisplatin-resistant ovarian cancer cells to cisplatin treatment. Mechanistically, the PI3K/AKT signaling pathway was activated in cisplatin-resistant cells, and this pathway would be affected by TTK expression. Furthermore, TTK was highly expressed in the tissues of ovarian cancer patients, especially those acquired resistance to cisplatin.

**Conclusions:**

Our study revealed that TTK may be a promising therapeutic target for cisplatin-resistant ovarian cancer.

**Supplementary Information:**

The online version contains supplementary material available at 10.1186/s13048-021-00884-z.

## Introduction

Ovarian cancer is one of the most common lethal gynecologic malignancy with the third morbidity rate and the first mortality rate. According to the cancer statistics, there are estimated approximately 200,000 new cases of ovarian cancer every year, with about 150,000 deaths attributed to the disease [1] all over of the world. Although the 5 years survival rate after early surgery is up to 80-90, 70% of the patients still have poor prognosis because it is usually diagnosed at advanced stages [2]. Surgery combined with cisplatin chemotherapy is the main treatment for malignant ovarian tumors. Most of the women diagnosed with advanced ovarian cancer will benefit from this treatment [3]. The pharmacological work of cisplatin is mediated by DNA binding, which can cause DNA damage, hinder DNA replication and induce cell death [4]. Unfortunately, the patients will be resistant to cisplatin-based chemotherapy after several cycles of treatment. Patients with cisplatin resistance usually have no responses to other chemotherapy drugs, and their non-progression lifetime is approximately 3-4 months with a median survival for less than 1 year. Therefore, it is urgent to elucidate the mechanism of cisplatin resistance and discover new therapeutic targets in the treatment of ovarian cancer.

Threonine and tyrosine kinase (TTK), an integral part of the spindle assembly checkpoint, works as a monitor mechanism for ensuring chromosomal separation [5]. High expression levels of TTK could result in the subsequent development of aneuploid tumors [6]. It has been reported that TTK is a predictor of poor prognosis in breast, lung, brain and colorectal cancer [7]. Meanwhile, recent studies have shown that TTK could mediate multiple drug resistance in lung cancer [1]. However, there was little information available regarding its role in ovarian cancer and cisplatin-resistance.

In this study, cisplatin-resistant ovarian cancer cell line A2780 (A2780cis) was established to explore the relationship between TTK expression and cisplatin-resistance. We clarified the mechanism of cisplatin-resistance through knockdown or inhibition of TTK expression. Furthermore, the related signaling pathways activated by TTK in cisplatin-resistant cell line were analyzed by RNA-sequencing (RNA-seq). The conclusions from cell experiments were further verified in ovarian cancer tissues. Thus, TTK may be a potential therapeutic target in patients with platinum-resistant ovarian cancer.

## Results

### TTK was up-regulated in cisplatin-resistant ovarian cancer cell line

**Fig. 1:**
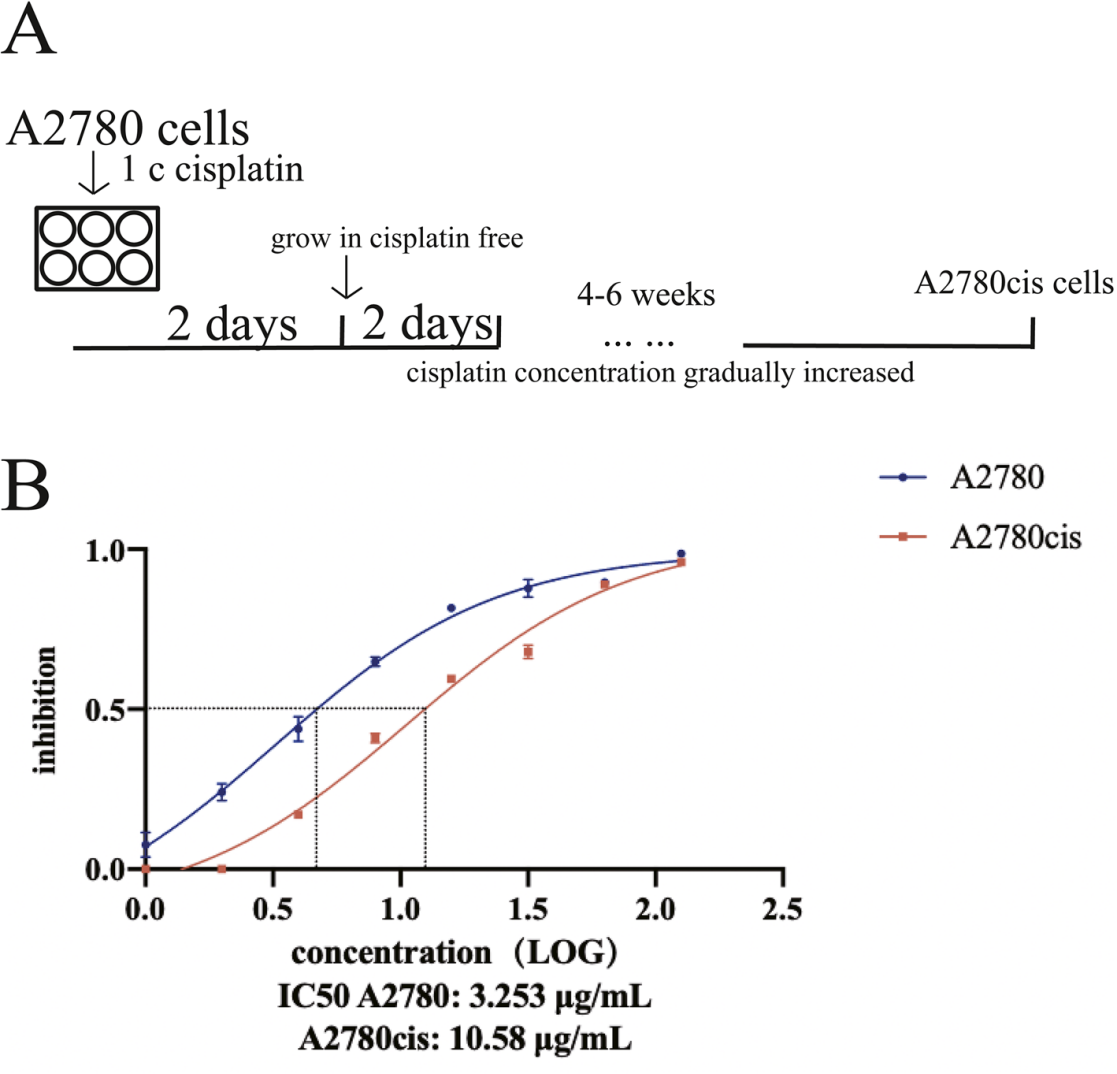
**A** The induction process of cisplatin-resistant ovarian cancer cell line A2780cis. **B** IC50 values of cisplatin in A2780 and A2780cis cells were analyzed by CCK-8 assay. The cisplatin concentrations used were as follows: 1 μg/mL, 2 μg/mL, 4 μg/mL, 8 μg/mL, 16 μg/mL, 32 μg/mL, 64 μg/mL, 128 μg/mL

**Fig. 2:**
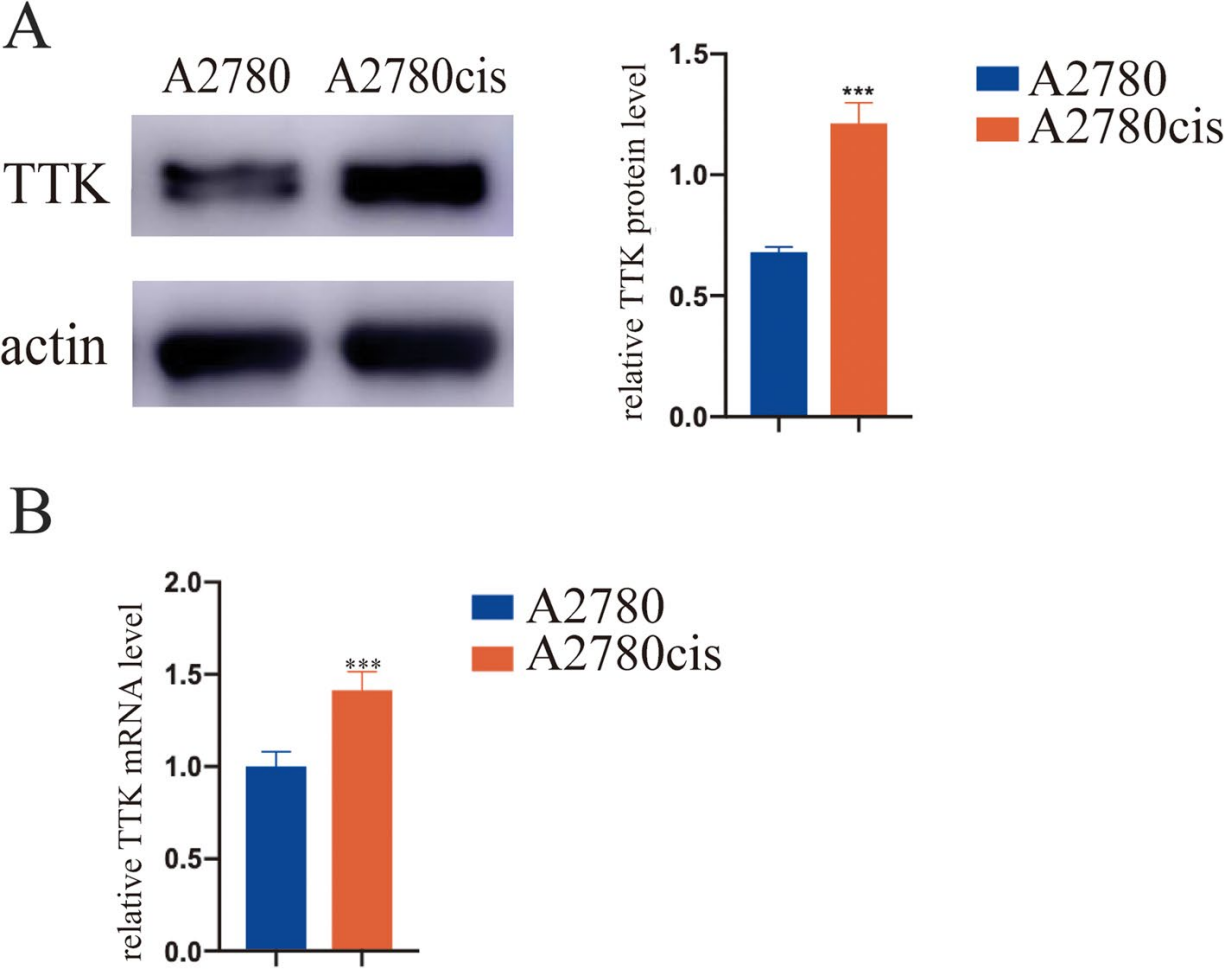
The up-regulation of TTK in cisplatin-resistant cell line was verified by **A** western blot and **B** quantitative real-time PCR compared with parental cells. ***P < 0.001

Cisplatin-resistant A2780 cell line was induced through continuous stimulation with cisplatin in vitro. A2780 cells were seeded in 6-well plate at 70-80% confluence and treated with 1 μg/mL cisplatin for 2 days. After 2 days’ culture, the cells were washed with PBS and recovered in medium without drug treatment for another 2 days. The above procedure was repeated for at least 4-6 weeks with gradually increased concentration of cisplatin (Fig. 1A). Then, we acquired a cisplatin-resistant A2780 cell line which could grow well in culture medium containing 10 μg/mL cisplatin. This cell line was named A2780cis. The cell viability was assayed after the treatment with 2-fold increasing concentrations of cisplatin for 48 h. The IC50 values of A2780 and A2780cis cells were 3.253 μg/mL and 10.58 μg/mL respectively (Fig. 1B). Therefore, A2780cis cell line is cisplatin-resistant and can tolerate more than three times higher concentration of cisplatin compared with parental A2780 cells. We found that the expression of TTK was increased in A2780cis cells at mRNA and protein levels (Fig. 2).

### Down-regulation of TTK expression could recover the sensitivity of A2780cis cells to cisplatin

**Fig. 3:**
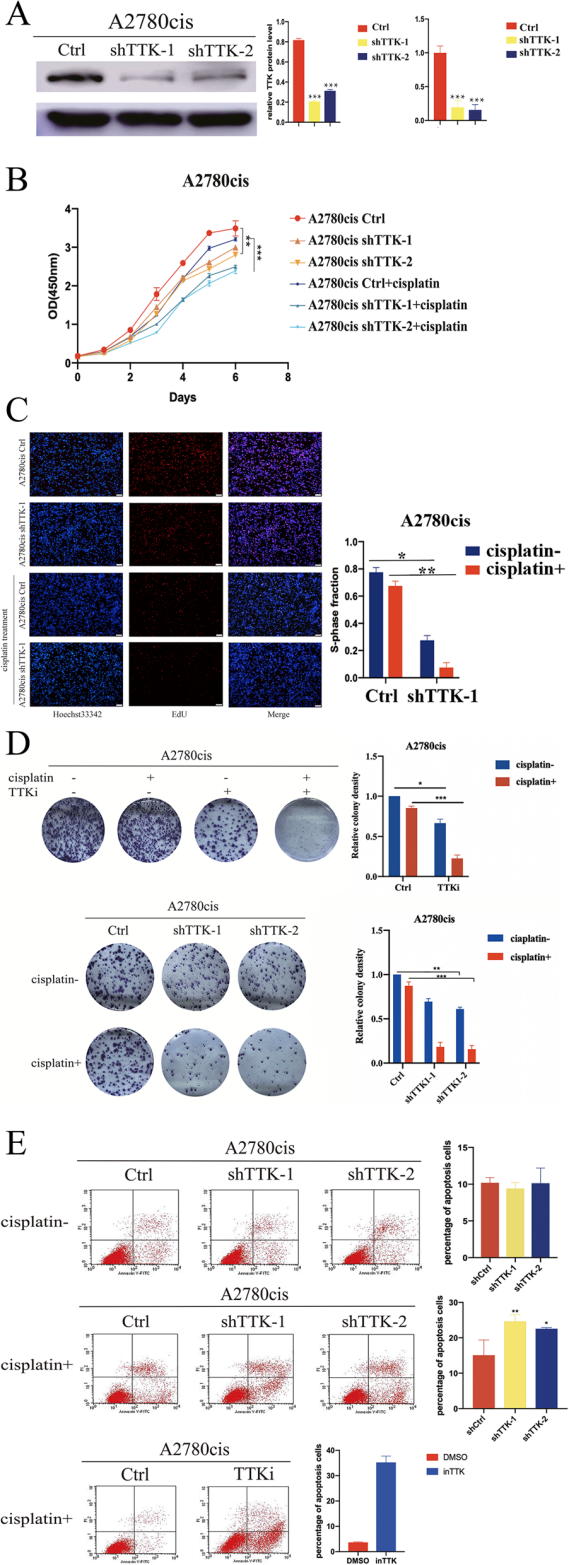
Knockdown of TTK expression in cisplatin-resistant cells would suppress cell proliferation and increase the drug sensitivity to cisplatin. **A** The knockdown of TTK in A2780cis cell line was verified by western blot and quantitative real-time PCR. **B** The cell proliferation of A2780cis cells with or without TTK knockdown was assayed by CCK8 kit. The growth of A2780cis cells with TTK knockdown was suppressed, especially in combination with cisplatin treatment. **C** DNA synthesis detection by EdU staining. The red fluorescence represents the ability of cell proliferation. **D** Colony formation assay showed that TTK inhibitor performed the same function as TTK knockdown. The clones were reduced when TTK was inhibited or up-regulated, especially in combination with cisplatin treatment. **E** Down-regulation of TTK or TTK inhibitor could significantly increase cell apoptosis rates with cisplatin treatment. *P < 0.05, **P < 0.01, ***P < 0.001

We found that the expression of TTK was increased in A2780cis cells at mRNA and protein levels (Fig. 2). To explore the relationship between TTK and cisplatin-resistance, TTK expression was down-regulated in A2780cis cells, and the knockdown efficiency was verified by Western blot and real-time PCR (Fig. 3A). As shown in Fig. 3B, knockdown of TTK protein inhibited the proliferation of A2780cis cells, and the influence was more obvious when the cells treated with cisplatin in culture medium. DNA synthesis detection by EdU (5-Ethynyl − 2′- deoxyuridine) staining also showed that the proliferation of A2780cis cells was suppressed after the down-regulation of TTK-. Especially, knockdown of TTK could improve the cisplatin-sensitivity of A2780cis cells (Fig. 3C). Meanwhile, down-regulation of TTK in A2780cis cells reduced the quantities of cell colony and promoted cell apoptosis. Moreover, TTK inhibitor could function the same as TTK knockdown (Fig. 3D, E). Therefore, we indicated that down-regulation or inhibition of TTK expression would suppress cell proliferation and increase cisplatin sensitivity of A2780cis cells.

### TTK inhibition relieved the resistance of A2780cis cells to cisplatin by inhibiting PI3K/AKT pathway

**Fig. 4:**
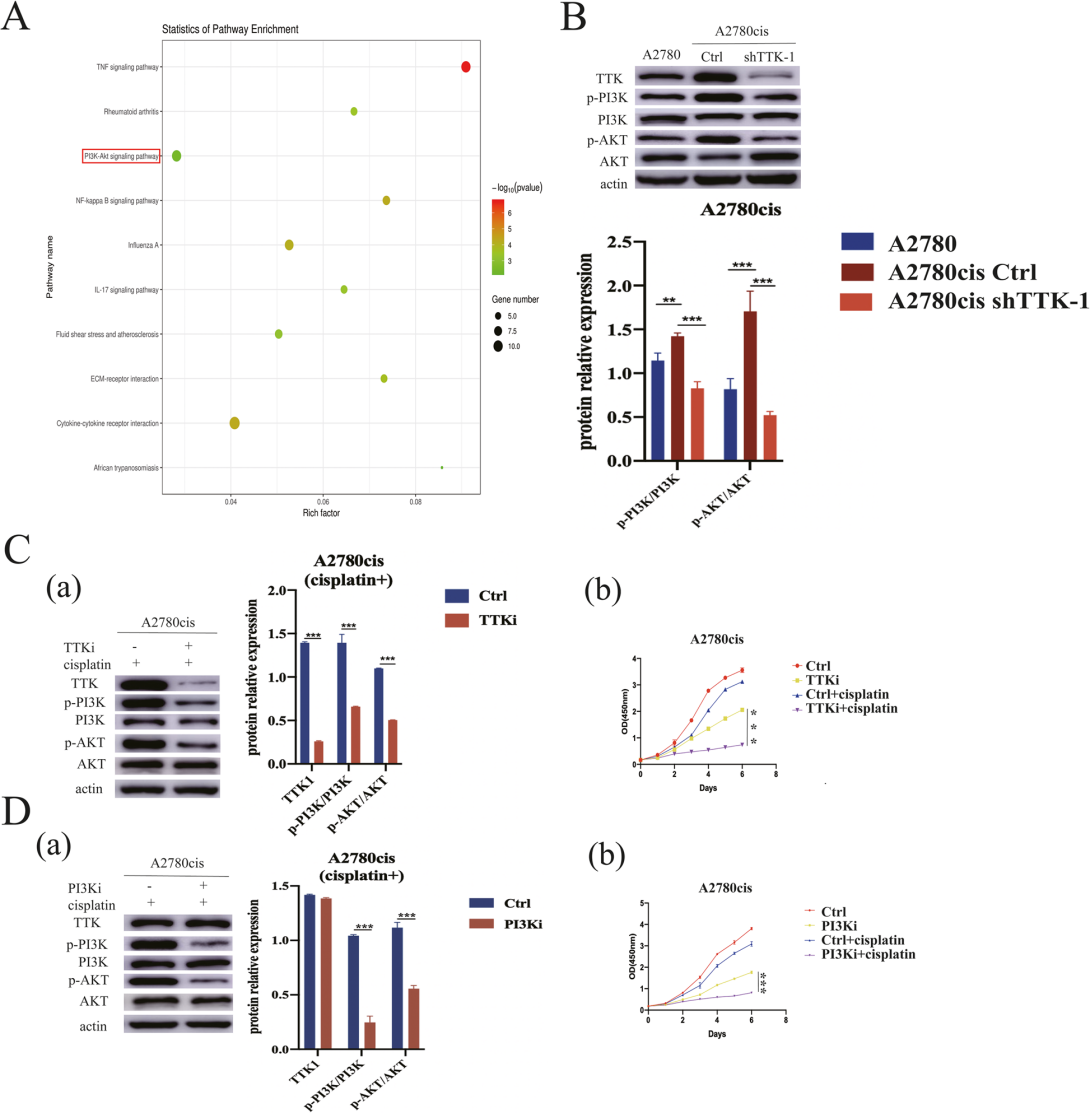
TTK activated PI3K/AKT signaling pathway. **A** RNA-seq results showed the top 10 differential genes between A2780cis cells with and without TTK knockdown. **B** The expression levels of p-AKT/AKT and p-PI3K/PI3K in A2780cis were higher than in A2780 cells, and their expression could be reduced when TTK was down-regulated in A2780cis cells. **C** A2780cis cells were treated with cisplatin and TTK inhibitor. (a) The phosphorylation levels of PI3K and AKT were inhibited using TTK inhibitor. (b) TTK inhibitor suppressed cell proliferation. **D** A2780cis cells were treated with cisplatin and PI3K inhibitor. (a) PI3K inhibitor would reduce the phosphorylation of PI3K and AKT without affecting TTK expression. (b) PI3K inhibitor suppressed cell proliferation. ***P < 0.001

The related signaling pathways activated by TTK were analyzed through RNA-seq. According to gene count and *P* value enriched by KEGG, we found the top 10 pathways differentially expressed in A2780cis cells with or without TTK knockdown (Fig. 4A). Among them, PI3K/AKT signaling pathway was reported to participate in cisplatin-resistance of ovarian cance [8]. Actually, both p-PI3K and p-AKT were up-regulated in A2780cis cells, suggesting that PI3K/AKT signaling pathway was activated when the cells were resistant to cisplatin (Fig. 4B). Thus, we guessed that TTK mediated cisplatin-resistance through activating the PI3K/AKT signaling pathway. As shown in Fig. 4B, knockdown of TTK could inhibit the phosphorylation of PI3K and AKT in A2780cis cells. The inhibitor of TTK or PI3K could significantly suppress the activation of the PI3K/AKT pathway and enhanced the cisplatin sensitivity of A2780cis cells (Fig. 4C, D). These results proved that TTK facilitated proliferation of cisplatin-resistant cells by activating PI3K/AKT signaling pathway.

### TTK was highly expressed in the tissues of ovarian cancer patients with cisplatin-resistance

**Fig. 5:**
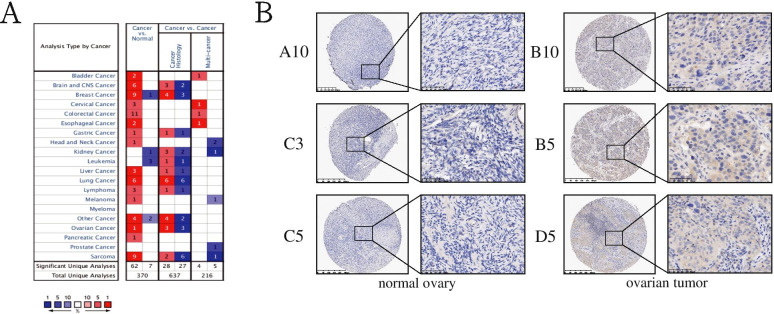
TTK was overexpressed in ovarian cancer patients. **A**The expression of TTK in Oncomine database. **B** Samples from tissue chip with different expression against TTK protein in ovarian tumor tissues and normal controls

**Fig. 6:**
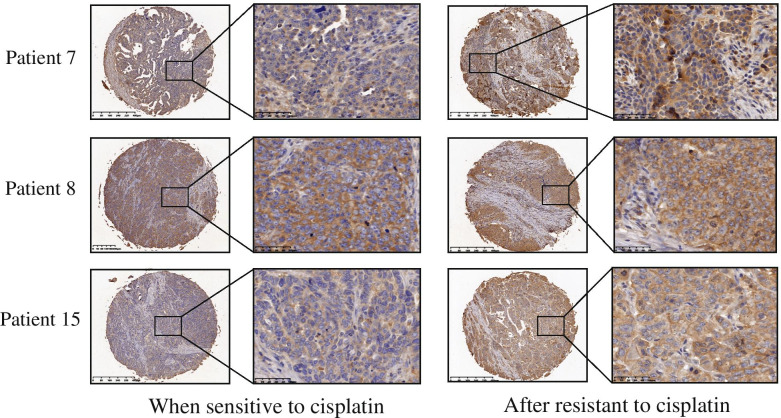
TTK was significantly overexpressed after ovarian cancer patients became cisplatin resistance. Samples from tissue chip with different expression against TTK protein in ovarian cancer patients before and after cisplatin resistance

Data from Oncomine (http://www.oncomine.org/) indicated that TTK was overexpressed in most types of cancer tissues than in normal tissues (Fig. 5A). We verified that the protein expression of TTK in ovarian tumor tissues was indeed higher than in normal controls by way of microarray chips- (*P* < 0.01) (Fig. 5B, Table 1). We further explored the expression of TTK in cisplatin-resistant ovarian cancer patients. Ovarian tumor tissues from 20 ovarian cancer patients were collected both before and after the cisplatin-resistance acquisition. We found that cisplatin-resistant tissues showed stronger TTK expression than the cisplatin-sensitive ones (Fig. 6, Table 2). Therefore, TTK may be a tumor promoter and cisplatin-resistance associated factor.

**Table 1:** The scores of TTK expression in normal ovary and ovarian tumor

	Age	Stage	IHC Scoring				*P*-value
			Negative expression (0)	Weak expression (2–4)	Moderate expression (5–6)	Strong expression (7–8)	
Normal ovary (*n* = 20)	39–79	/	20	/	/	/	*P* < 0.0001
Ovarian tumor (*n* = 20)	34–66	IIIC-IV	/	7	11	2

**Table 2:** The scores of TTK expression in ovarian cancer patients before and after resistant to cisplatin

	IHC Scoring				*P*-value
	Negative expression (0)	Weak expression (2–4)	Moderate expression (5–6)	Strong expression (7–8)	
When sensitive to cisplatin (*n* = 20)	0	9	7	4	*P* < 0.001
After resistant to cisplatin (*n* = 20)	0	0	3	17	

## Discussion

Combination of surgery and cisplatin-based chemotherapy is the gold standard for the treatment of ovarian cancer. However, after short-term treatment, cisplatin resistance often occurs, which greatly weakened the therapeutic effect [9]. Cellular resistance to cisplatin occur through multiple mechanisms which can be divided into the following categories: a) reducing cross-linking in the DNA chain, such as preventing the drug intake and accelerating the drug efflux [10–12]. b) increasing the efficiency of DNA repair [13]. c) promoting cell survival through changing the signaling pathway that affects the tumor apoptosis [14, 15]. However, the key factors associated with chemotherapy resistance still need to be explored.

The protein kinase TTK (also known as Monopolar spindle 1, Mps1), a dual specificity kinase, is involved in the formation of mitotic checkpoint complex, regulation of cytokinesis, response to DNA damage, and facilitation of proper chromosome alignment [16]. TTK is hardly detectable in normal organs except the testis and placenta. However, high levels of TTK are found in many types of human malignancies. Overexpression of TTK promotes tumor growth in prostate cancer [17], breast cancer [18] and colon cancer [19]. Thus, we guess that TTK inhibitor in combination with antimitotic cancer drugs will enhance their efficacy and potentially overcome resistance.

In this study, we explored the possibility of sensitivity-reacquisition to cisplatin in ovarian cancer cells through targeting for TTK. Actually, TTK expression was reduced when ovarian cancer cells were treated with cisplatin, indicating that TTK may serve as a chemo-therapeutic target (Fig. S1). Cisplatin-resistant cell line A2780cis was established by continued stimulation with gradually increased concentration of cisplatin. In cisplatin-resistant ovarian cancer cells, TTK was up-regulated compared with the parent tumor cells. Meanwhile, the overexpression of TTK in cisplatin-resistant patients was verified by immunohistochemistry. In the present study, we focus not the reason to cause resistance, but on the way to overcome resistance. Therefore, it is unclear whether the overexpressed TTK leads to cisplatin resistance or the cisplatin-resistant cells cause TTK overexpression. In our study, tumor cells will become resistant to chemotherapy by several molecular mechanisms during the drug treatment. To tolerate genomic instability and aneuploidy, the cisplatin-resistant cells will develop adaptive mechanisms. The spindle assembly checkpoint (SAC) is the main mechanism that maintains chromosomal stability through the cell division. TTK is a critical SAC component, which can promote the formation of mitotic checkpoint complex. Overexpression of TTK may be one of the important mechanisms for resistant tumor cells to live with aneuploidy.

The results of the RNA-seq prove that TTK may have an impact through the change of p53 to affect the sensitivity of ovarian cancer to platinum drugs. p53 is an important tumor suppressor gene, involved in important biological processes such as cell differentiation, cell apoptosis, DNA repair, and cycle regulation [20]. The p53 signal pathway is an important way for platinum drugs to cause tumor cell DNA damage. Studies have shown that p53 protein aggregation damages the normal transcription of p53 gene and its apoptotic functions in different types of tumor cells, and can promote platinum resistance in ovarian cancer [21].

JAK/STAT signal transduction pathway is the common pathway of many cytokines, and it is widely involved in the processes of cell proliferation, differentiation, apoptosis and inflammation [22]. JAK/STAT pathways [22, 23] play an important role in tumor occurrence, development and drug resistance, and may become a new target for tumor treatment.

PI3K/AKT/NF-κB signal pathways [24, 25] are involved in tumor occurrence, development, invasion and metastasis. Some studies have shown that PI3K inhibitors are used for treatment of several human tumors, such as ovarian cancer [26], breast cancer [27], and leukemia [28]. In addition, it was also reported that activation of AKT was related to cisplatin-resistance in ovarian cancer [29]. We further clarified that the activated signaling pathway by TTK was PI3K/AKT pathway, which played an important role in proliferation, migration, invasion and chemotherapy resistance [25].

We verified that inhibition of TTK expression could prevent the activation of PI3K/AKT pathway in cisplatin-resistant cells. However, the specific mechanisms for TTK to activate PI3K/AKT pathway need to be explored.

## Conclusions

Our study demonstrated that TTK was overexpressed in ovarian cancer, and was further up-regulated in cisplatin-resistant cells. Knockdown or inhibition of TTK expression could significantly suppress cell proliferation and overcome cisplatin resistance through inhibition of PI3K/AKT pathway. These findings revealed that TTK emerged as a potential therapeutic target in ovarian cancer, especially in combination with chemotherapy.

## Materials and methods

### Cell culture

A2780cis and A2780 cells were cultured in RPMI-1640 medium (Gibco, USA) containing 10% fetal bovine serum (Gibco) and 1% penicillin-streptomycin (Gibco). HEK 293 T cells were cultured in DMEM (Gibco) containing 10% fetal bovine serum (Gibco) and 1% penicillin-streptomycin (Gibco). All cells were cultured at 5% CO_2_ in a 37 °C incubator.

### Tissue samples

Twenty ovarian cancer tissues and 20 normal ovarian tissues were obtained from patients undergoing resection of ovarian cancer or other diseases at the Department of Ovarian Surgery, Fudan University Shanghai Cancer Center. After the histopathologic diagnosis was defined by professional pathologists, the corresponding tissue samples were collected for TTK detection. Written informed consent was available from all patients.

### Inhibitors and antibodies

The inhibitors used in this study were TTKi (5 μM, MPI-0479605, Selleck) and PI3Ki (1 μM, BYL719, Selleck). All compounds were dissolved in DMSO at a concentration of 10 mM and stored at − 80 °C before use. The cells were treated with inhibitors for 3 days while the control cells were treated with DMSO only. The antibodies used in western blot were as follows: TTK (sc-376,842, 1:500 dilution, SANTA CRUZ, USA), β-actin (60008-1-lg, 1:3000 dilution, Proteintech Group, China), AKT (4685 T, 1:1000 dilution, CST, MA, USA), p-AKT (4056, 1:1000 dilution, CST, MA, USA), PI3K (4257, 1:1000 dilution, CST, MA, USA), p-PI3K (4228, 1:1000dilution, CST, MA, USA). Western blot was performed according to a previous publication [25]. The protein bands were analyzed by Image J software (NIH, USA).

### Immunohistochemistry and evaluation

TTK was detected with a mouse monoclonal antibody at a dilution of 1:50(sc-376,842, Santa Cruz Biotechnology, CA, USA). IHC stain and scoring criteria for TTK was described as our previous publications [30].

### TTK knockdown in tumor cells

The short hairpin RNA (shRNA) was designed with the manufacturer’s RNAi Designer program, and the sequences are shown in Table 3. The recombined pLKO.1 plasmids along with packing plasmids psPAX2 and pMD2.G (Addgene) were transfected into HEK293T cells using Lipofectamine 3000 transfection reagent (Invitrogen, CA, USA). After 48 h, virus supernatant was collected and ovarian cancer cells were infected with the virus in the presence of 8 μg/mL polybrene (Sigma, USA). The stable transfected cells (shCtrl, shTTK-1, shTTK-2) were selected for 3-5 days using 2-10 mg/mL puromycin (Sigma-Aldrich, MO, USA).

**Table 3:** Sequences of shRNA against TTK for transfection

Name	Sequence
shCtrl (scrambled sequence)	5′-CCTAAGGTTAAGTCGCCCTCG-3′
shTTK-1	5′-GATAAGATCATCCGACTTTAT-3′
shTTK-2	5′-GCACAATTTGAACTGTCACAA-3′

### Colony formation assay

Cells were seeded into 6 well-plates at a density of 1000 cells/well and incubated for 10-14 days until visible clones were appeared. Colonies were stained with 0.5% crystal violet and a microscope was used to determine the quantity of colonies.

### Quantitative real-time PCR (qRT-PCR)

Total RNA was isolated from cultured cells using Trizol reagent (Invitrogen, USA) according to manufacturer’s instructions. Total RNA was reversely transcribed into complementary first strand DNA using RT kit (TaKaRa, Japan). PCR was performed with SYBR Green PCR Kit (Thermo Fisher Scientific, Rockford, USA) using an ABI PRISM Detection System (Applied Biosystems, Life Technologies). All samples were performed in triplicate and normalized to GAPDH levels. The specific primers used are listed in Table 4.

**Table 4:** Primer sequences for real-time qPCR

Gene	Sequence (5′-3′)
TTK	F: TCCCCAGCGCAGCTTTCTGTAGA
	R: CCAGTCCTCTGGGTTGTTTGCCAT
GAPDH	F: GGAGCGAGATCCCTCCAAAAT
	R: GGCTGTTGTCATACTTCTCATGG

### DNA synthesis measuring

EdU (5-Ethynyl-2′-deoxyuridine) is a thymidine analogue, which can replace thymine (T) to be incorporated into the replicating DNA molecule during cell proliferation. EdU kit (Cell-Light EdU Apollo 488 In Vitro Imaging Kit, RiboBio, Guangzhou, China) was used to measure DNA synthesis according to the manufacturer’s instructions. After incubation with 50 μM EdU for 2 h at 37 °C, cells were washed with PBS and then fixed with 4% paraformaldehyde for 30 min, followed by incubation with 2 mg/mL glycine for 5 min. Nucleus was stained with DAPI.

### Cell proliferation detection

Cells were seeded into 96-well plates and treated with different concentrations of cisplatin (0-128 μg/mL). Cell proliferation was detected using cell counting kit-8(Dojindo, Japan) according to the manufacturer’s instructions. Optical density (OD) value was detected at 450 nm on a microplate reader. Viability was measured using the following formula: relative viability (experimental absorbance – background absorbance) / (untreated controls absorbance-background absorbance) *100%. The IC50 values of cisplatin were calculated using nonlinear regression (GraphPad Prism v3.0, GraphPad software).

### Cell apoptosis detection

The cells were harvested and re-suspended in 0.5 ml binding buffer, then incubated with Annexin-V fluorescein isothiocyanate/PI dual stain (BD Biosciences) for 15 min and finally determined by flow cytometry.

### RNA sequencing analysis

The total RNA in A2780cis cells and A2780cis cells with TTK knockdown cells were extracted using the Trizol (Invitrogen, USA) and then were sent to Mingma Biotechnology Company (Shanghai) for sequencing. Sequencing results were further analyzed using R language.

### Statistical analysis

Statistical analysis was performed using SPSS 24.0 software. The data were compared between two groups by an independent Students’ *t*-test. The positive proportion of TTK expression was analyzed using chi-square test. Differences were considered statistically significant when *P* < 0.05.

## Supplementary Information

**Additional file 1: Fig. S1.** (A) Tissue chip of TTK expression in ovarian tumor tissues and normal controls. (B) Tissue chip of TTK protein in cisplatin-sensitive and cisplatin-resistant ovarian tumor tissues. (C) TTK expression was reduced after cisplatin treatment in A2780 cells.

## Data Availability

The data used and analyzed during the current study are available from the corresponding author on reasonable request.
