# Supplementary figures and images for: TTK is a potential therapeutic target for cisplatin-resistant ovarian cancer
Source: J Ovarian Res. 2021 Oct 2;14:128. doi: 10.1186/s13048-021-00884-z (PMC8487155; doi:10.1186/s13048-021-00884-z)

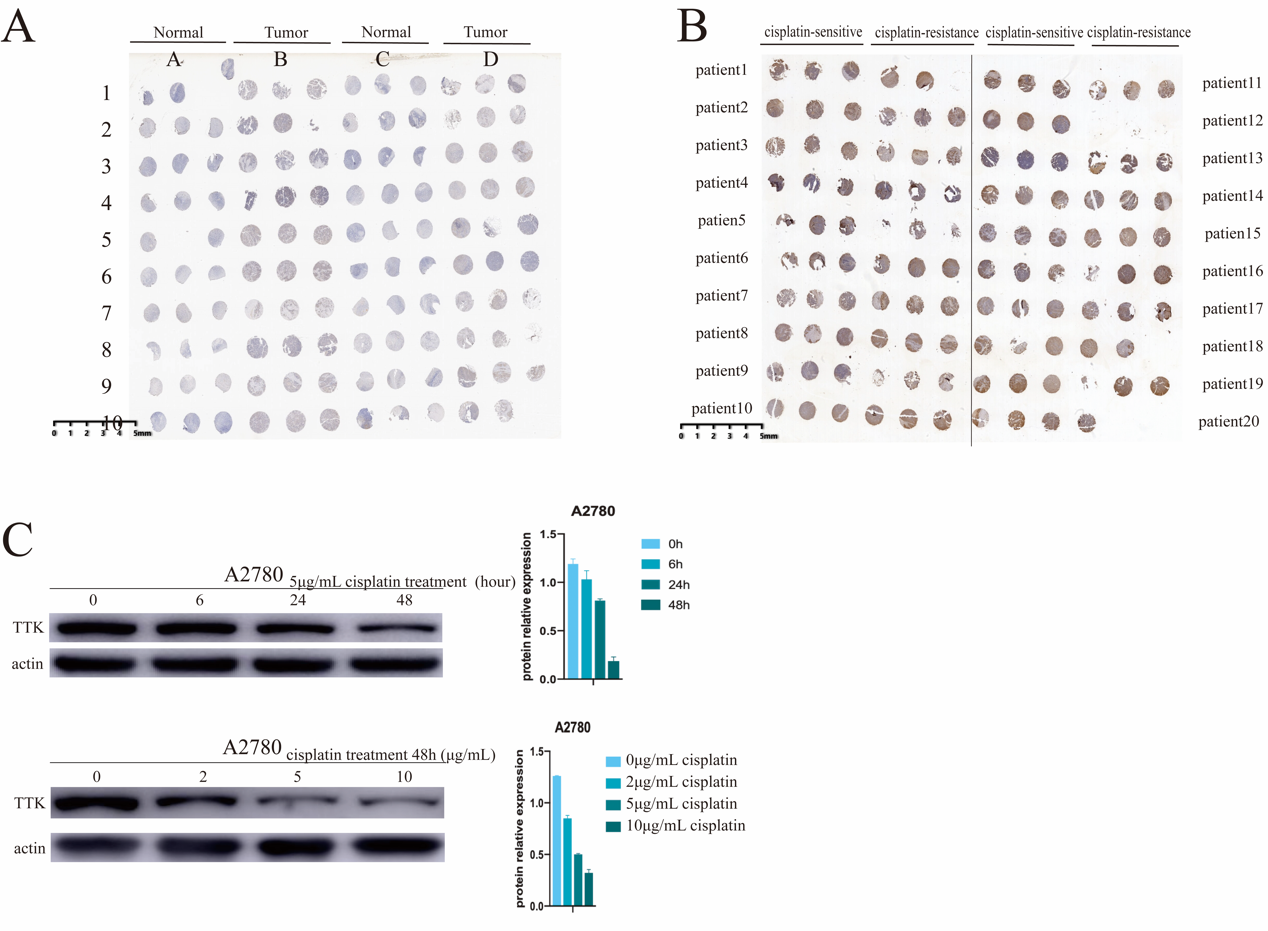


Fig. S1

Supplement: Supplementary file 1 — Additional file 1: Fig. S1. (A) Tissue chip of TTK expression in ovarian tumor tissues and normal controls. (B) Tissue chip of TTK protein in cisplatin-sensitive and cisplatin-resistant ovarian tumor tissues. (C) TTK expression was reduced after cisplatin treatment in A2780 cells. [file 13048_2021_884_MOESM1_ESM.docx]
